# Evolution in the Practice of Pediatric Endoscopy and Sedation

**DOI:** 10.3389/fped.2021.687635

**Published:** 2021-07-14

**Authors:** Conrad B. Cox, Trevor Laborda, J. Matthew Kynes, Girish Hiremath

**Affiliations:** ^1^Division of Pediatric Gastroenterology Hepatology, and Nutrition, Monroe Carell Jr. Children's Hospital at Vanderbilt, Nashville, TN, United States; ^2^Division of Pediatric Gastroenterology, Hepatology, and Nutrition, University of Utah Primary Children's Hospital, Salt Lake City, UT, United States; ^3^Division of Pediatric Gastroenterology, Hepatology, and Nutrition, Baylor College of Medicine, Children's Hospital of San Antonio, San Antonio, TX, United States; ^4^Department of Anesthesiology, Monroe Carell Jr. Children's Hospital at Vanderbilt, Nashville, TN, United States

**Keywords:** gastrointestinal endoscopy, sedation administration, artificial intelligence, pediatric, history, ERCP

## Abstract

The fields of pediatric gastrointestinal endoscopy and sedation are critically important to the diagnosis and treatment of gastrointestinal (GI) disease in children. Since its inception in the 1970s, pediatric endoscopy has benefitted from tremendous technological innovation related to the design of the endoscope and its associated equipment. Not only that, but expertise among pediatric gastroenterologists has moved the field forward to include a full complement of diagnostic and therapeutic endoscopic procedures in children. In this review, we discuss the remarkable history of pediatric endoscopy and highlight current limitations and future advances in the practice and technology of pediatric endoscopy and sedation.

## Introduction

Pediatric endoscopy came into existence nearly 170 years after Dr. Phillip Bozzini developed the first “endoscope” in 1805 known as the “Lichleiter” candle (1). At that time and throughout the 1800s, endoscopy was plagued by inadequate and even dangerous methods of combustible lighting, but Thomas Edison's electric light bulb quickly resolved this issue in the 1880s. Despite improved illumination, endoscopes remained limited by poor visualization and rigidity which prevented access to deep body cavities like the proximal colon and duodenum (2). Then, in 1958, Dr. Hirschowitz famously described his clinical experience with the first fiberscope (3). This technology paved the way to modern flexible endoscopes by incorporating bundled glass fibers to transmit light and images. By the 1970s fiberscopes had become widely available, but their use in small children remained limited because of the problem of miniaturizing the equipment. During this time, smaller 5.2 mm fiber bronchoscopes were being used in children, but these were not suitable for examination of the gastrointestinal tract because of poor image quality, limited angulation, and lack of suction or insufflation (4).

In 1969 the Hopkins rod-lens system permitted miniaturization of the endoscope. This revolutionary system paved the way for development of the pediatric specific fiber endoscope (5, 6). In the ensuing two decades fiberoptics gave way to the charge-coupled device (CCD) video endoscope which was introduced by Welch Allyn in 1983 (7). A CCD allowed real time image display on video monitors which transformed the field and art of endoscopy. Over the past 40 years, advances in biomedical technologies have led to real-time visualization of the luminal features through high-definition images, the current industry standard. The obstacles of lighting, optics, and maneuverability have been mostly conquered with today's technology, but the design of the gastrointestinal endoscope remains relatively unchanged leaving multiple problems yet unsolved including procedural discomfort, loop related perforation, difficult sterilization, and subtotal examination of the GI tract (8). Because of the inherent risks and discomfort associated with the current endoscope design, deep sedation or general anesthesia are essential.

## Sedation in Children Undergoing Gastrointestinal Endoscopy

Sedation for pediatric gastrointestinal endoscopy is an important component for patient comfort and procedural success. Pediatric developmental and physiologic considerations, however, require a specialized approach to avoid serious complications. Although the occurrence of serious complications from pediatric procedural sedation performed by experienced practitioners in a culture of safety is <2% (9), life-threatening events during sedation still occur (10). The trend of increasing use of sedation for diagnostic and therapeutic procedures by a wider array of providers, including pediatric hospitalists (9), underscores the need for continued vigilance around sedation safety.

Cardiopulmonary and sedation-related adverse events may account for up to 60% of periprocedural complications from pediatric endoscopy (11). Patient groups at elevated risk for cardiopulmonary and sedation-related adverse events have been identified through analysis of large pediatric outcomes databases (12, 13). These high-risk groups include infants younger than 1 year and children with significant congenital comorbidities, such as congenital heart disease, cystic fibrosis, muscular dystrophy, or acquired comorbidities, such as obesity and acute upper respiratory tract infection (11). Preprocedural assessment for these and other conditions affecting hemodynamic stability, airway management and aspiration risk and assigning an American Society of Anesthesiologists (ASA) physical status score are a standard recommendation of the American Academy of Pediatrics (10).

Given the anxiety and discomfort associated with pediatric endoscopy, deep sedation or general anesthesia administered by a dedicated provider or anesthesiologist are typically required (14). Sedation is often achieved with a combination of fentanyl, meperidine, midazolam, or ketamine. Endoscopist performed sedation is technically difficult, time consuming, and may increase the risk for adverse cardiopulmonary events. Therefore, multiple authors suggest dedicated anesthesiologists or carefully selected sedation teams in line with national legislation and institutional regulations to perform endoscopic sedation (15, 16). In the interest of patient safety, current guidelines for deep sedation and/or general anesthesia continue to require at least two individuals present including a skilled observer independent of the procedure itself with training and credentialing in sedation and advanced airway skills capable of patient rescue during life-threatening emergencies (10).

Increased attention has focused on expanding options for non-anesthesiologist-administered sedation including with propofol. Propofol is a potent amnestic and hypnotic agent with rapid onset and short duration allowing for rapid titration to a targeted depth of sedation without gastrointestinal side effects. Its primary disadvantage its narrow therapeutic window and ease of moving quickly between levels of sedation into general anesthesia with potential airway, respiratory and hemodynamic compromise. With proper training and institutional support, usually aligned with practice recommendations from the ASA (15), credentialing pathways have emerged for deep sedation using propofol in adult gastroenterology practice based on high-level evidence (16). Building on reports of propofol administration by non-anesthesiologists for pediatric procedural sedation (17), researchers have continued to establish a favorable safety profile for a team-based approach to sedation with propofol in pediatric endoscopy even compared with general anesthesia (18). This issue remains unresolved, however, and safety considerations when using propofol remain significant as the largest database of pediatric procedural sedation outcomes reported increased risk of adverse events, especially airway events, in sedation using propofol alone or in combination with other agents (9).

### Putting It Into Practice

- Pediatric sedation for gastrointestinal endoscopy results in rare but sometimes serious adverse events and requires a specialized approach. It is important to determine sedation risk based on the patient's profile and their ASA physical status score.
- Pediatric sedation is technically challenging and requires the expertise of pediatric anesthesiologists or an approved hospitalist led sedation team in accordance with ASA guidelines, institutional regulation, and applicable legislation. Endoscopist performed sedation is not advised as it results in decreased patient satisfaction, increased procedure time, and leads to higher risk for cardiopulmonary adverse events.

## Common Endoscopic Procedures in Children

As with anesthesia, selection of the proper endoscopic tools in pediatric endoscopy is necessary for procedural success and patient safety. Endoscopes are presently manufactured in a range of sizes to permit access into the gastrointestinal tract of children. Diagnostic endoscopy includes the acquisition of endoscopic images and sampling of mucosal tissue and includes both esophagogastroduodenoscopy (EGD), and colonoscopy. These procedures can be performed by an adequately trained pediatric gastroenterologist with an appropriately sized endoscope.

Depending upon the pathology, therapeutic procedures are sometimes indicated. A range of endoscopic therapies are available but only a handful are typically employed by general pediatric gastroenterologists. Some of these procedures include stricture dilation, variceal ablation, polypectomy, foreign body management, hemostatic therapy, and transnasal endoscopy. However, options for some of these remain limited in ultrathin pediatric gastroscopes. These scopes, including trans-nasal endoscopes, range from 4.9 to 5.9 mm in diameter, contain a single 2.0–2.4 mm working channel and are requisite in children weighing <5 kg. Due to these size restrictions some therapies like endoscopic balloon dilation, use of large retrieval devices, and application of topical hemostatic agents are not possible (17, 18).

Slim gastroscopes have an insertion diameter of 7.8–9.0 mm and are typically used in children weighing <10–15 kg. Standard gastroscopes range from 9.0 to 10.0 mm in diameter and are useful in children weighing more than 20 kg (18). The primary advantage of these endoscopes compared with ultrathin models is the 2.8 mm working channel which supports most therapeutic instruments including balloon dilators, retrieval devices, polypectomy snares, and hemostatic therapies.

Esophageal stricture dilation is an important procedure for pediatric endoscopists (19). Balloon dilators come in various sizes and are important tools because of their ability to create an even distribution of circumferential pressure on a stricture. Balloon dilation is often advantageous over bouginage because of several key features including endoscopic and fluoroscopic real-time evaluation of balloon placement and stricture reduction, wire guided balloon placement in difficult-to-reach locations, and lower rates of post-procedural pain, although both have similar safety profiles (20, 21). This therapy is only available when using endoscopes with 2.8 mm working channels so alternative methods must be employed in smaller patients (22).

Management of gastrointestinal foreign bodies in children is a unique and important aspect of pediatric gastroenterology. Some solid ingestions like esophageal button battery, multiple magnets, or sharps necessitate rapid resolution (23). In addition to emergent ingestions, other objects requiring endoscopic retrieval may simply be too large to pass a child's lower esophageal sphincter or pylorus. Various retrieval devices are manufactured to fit in standard gastroscopes, and it is important for endoscopy units to maintain a stocked armamentarium of this equipment.

Significant gastrointestinal bleeding is rare in the pediatric population, but this represents an important indication for endoscopy. Appropriate endoscopy unit planning and stocking is required to manage these events effectively. Hemostatic therapies broadly include, mechanical, thermal, topical, and injection methods (24). Ultrathin pediatric scopes are unable to support the use of topically applied powders including Hemospray (TC-325, Cook Medical, Bloomington, Indiana, United States) and Endoclot (EC, Micro-Tech Europe, Düsseldorf, Germany), as well as mechanical clips (18, 25). Available hemostatic therapies for ultrathin scopes include 22–25 g injection needles, argon plasma, and thermal contact devices. This again underscores the importance of maintaining a variety of endotherapies for use in both standard and ultrathin sized endoscopes.

TNE offers pediatric endoscopists the ability to perform non-sedated endoscopy in the clinic setting. However, free-standing gastroenterology offices may encounter logistical difficulties related to scope reprocessing. TNE has historically been used in adult patients but is garnering attention in pediatrics in part because of disorders like eosinophilic esophagitis (EOE) which require serial endoscopies. A major benefit of TNE is that it overcomes the need for sedation and can be performed within the clinic setting (26) thereby reducing cost, time, and sedation related adverse events. Anxiety surrounding non-sedated TNE may be mitigated by the novel use of virtual reality video goggles, a strategy that has been successful in children as young as 6 years (27).

### Putting It Into Practice

- The smaller working channel found in ultrathin endoscopes can present therapeutic challenges for certain disease states.
- Pediatric endoscopy centers should maintain appropriate quantities of endoscopic tools for foreign body management and hemostasis in both standard and ultrathin pediatric scopes.
- Limit accidental unpackaging of inappropriate sized equipment by clearly designating scope size requirements.
- To successfully employ TNE within the gastroenterology clinic it is important to consider the logistics of daily scope reprocessing. TNE is beneficial for patients who require serial esophagoscopy and it can be helpful to utilize non-pharmacologic methods for anxiolysis such as virtual reality goggles.

## Advanced Endoscopic Procedures in Children

Advanced endoscopic procedures including endoscopic ultrasound (EUS), endoscopic retrograde cholangiopancreatography (ERCP), and per-oral endoscopic myotomy (POEM) are becoming more widely available to children, but many pediatric centers still cannot offer these therapies. These therapies provide minimally invasive solutions for patients with illnesses that previously would have been surgically managed (28, 29). A recent published survey of North American pediatric gastroenterologists showed that 72% of respondents believed their institutions' arrangement for advanced endoscopic procedures was inadequate (30). This discrepancy is the result of an historically low supply of advanced pediatric endoscopists and pediatric case load in addition to scare training options (31).

ERCP in pediatrics has been increasing steadily over the past 20–30 years and has shifted from a diagnostic to a therapeutic procedure. For most patients weighting >10 kg a standard adult duodenoscope can be utilized. However, for smaller patients a pediatric duodenoscope must be used (32). ERCP is technically demanding with higher complication rates than standard endoscopy and proper patient selection is key in preventing complications. Increasing evidence continues to demonstrate its safety and efficacy in pediatrics (33–36). ERCP is performed in pediatrics primarily for pancreaticobiliary indications such as: biliary obstruction, pancreatic ductal stones, acute recurrent and chronic pancreatitis, pancreas divisum, choledochal cysts, trauma, and sphincter of Oddi dysfunction. The major limitations of pediatric ERCP continue to be duodenoscope size, lack of pediatric specific instruments and endoscopes and lack of adequate training.

Endoscopic ultrasound (EUS) has diagnostic and therapeutic relevance in pediatrics along with promising patient safety data (37–39). EUS is used in idiopathic recurrent pancreatitis, pancreatic pseudocysts, walled-off necrosis, cyst-gastrostomy creation, cyst-duodenoscopy, fine needle aspiration and biopsy, suspected choledocholithiasis, celiac plexus block, submucosal lesions, and congenital malformations (40, 41). An advantage of EUS compared with other radiologic exams is its ability for precision tissue sampling. EUS can further discriminate the appropriateness of ERCP in patients whom the diagnosis of choledocholithiasis is unclear (42). Patient-scope size mismatch can again present challenges in pediatrics as the weight cut-off for EUS is typically >15 kg.

POEM has become an important procedure for the management of pediatric achalasia and is being performed by both surgeons and gastroenterologists. Achalasia has long been managed with pneumatic balloon dilation and surgical correction but a meta-analysis from 2019 demonstrated the superiority of POEM for all three achalasia subtypes (43). POEM was first performed by Dr. Haruhiro Inoue in 2008 (44) and since then, several pediatric case series have reported clinical success rates of 90–100% with only minor complications (45, 46).

The future of pediatric advanced endoscopy is bright with new advances in training, pediatric specific endoscopes, and instrument development. However, to continue to advance the field of pediatric advanced endoscopy it is crucial to maintain a collegial relationship with our adult GI colleagues (30).

### Putting It Into Practice

- Pediatric centers are frequently unable to provide advanced endoscopic procedures, however, adult advanced gastroenterologists may be able to help manage some patients. Establish and maintain a collegial relationship with local advanced endoscopists who are willing and able to care for pediatric patients.
- Pediatric endoscopists may acquire advanced endoscopic skills through formal or informal training programs in the United States and globally. This will help meet needs at institutions where advanced procedures are limited.
- EUS should be performed in cases where choledocholithiasis is uncertain as it frequently avoids unnecessary ERCP.
- POEM has become an important treatment option for pediatric achalasia and is being offered at numerous centers. POEM can be considered for first line treatment but may also be considered after unsuccessful surgical myotomy.

## Complications Related to Endoscopic Procedures in Children

Adverse events (AEs) related to pediatric endoscopy are rare but high-quality large-scale data remains scant. However, recent publications and clinical practice guidelines have helped inform practicing gastroenterologists and guide quality improvement measures within endoscopy units.

Until recently, the largest studies detailing AEs in pediatric endoscopy came from retrospective multi-center datasets. The 2006 PEDS-CORI report acquired data during or immediately following pediatric gastroduodenoscopies and cited a 2.3% (1.6% anesthesia related and 0.7% endoscopy related) overall AE rate with no deaths or perforations (47). This study likely missed late presenting AEs. A report from the Pediatric Hospital Information System (PHIS) in 2017 described a 0.7% 5-day readmission rate following diagnostic EGD and colonoscopy. However, only 6.6% of these cases required inpatient treatment. Despite its inability to describe specific AEs, this report importantly noted that minority race, female sex, and complex chronic conditions were factors more commonly associated with readmission (48). PHIS data revealed an overall therapeutic procedure complication frequency (0.74%) and mortality rate (0.1%) and identified higher risk for readmission following variceal ablation and stricture dilation compared with other procedures (49).

A more recent study prospectively evaluated AEs within the 72 h following endoscopy over 4 years and reported a 2.6% cumulative AE rate from all diagnostic and therapeutic endoscopies. Medically significant AEs related to infection, bleeding, and perforation were encountered in only 0.28%, and therapeutic procedures accounted for most of these cases (50). This study improved our understanding of the adverse event profile following endoscopy by defining specific events within the 72 h following endoscopy.

Despite the low rate of serious AEs, these remain a concern and warrant attention. A review from 2018 cited various studies which identified endoscopist experience, pre-procedural assessment, identification of appropriate equipment, and CO2 insufflation as the most frequently proposed counter measures to reduce procedural complications (51). The North American Society of Pediatric Gastroenterology, Hepatology, and Nutrition (NASPGHAN) endoscopy committee made formal quality improvements recommendations for endoscopy units to include a preoperative assessment designed to identify high risk patients (11) and for individual institutions to track AEs (52). It is imperative that pediatric gastroenterology fellows receive adequate hands-on experience during training to develop proper technique, but also that they readily understand the indications and proper use of all endoscopic equipment.

Finally, it is important that post-procedural complications including fever and abdominal pain are handled appropriately. In most cases these symptoms are unrelated to serious AEs and providing reassurance can help allay caregiver anxiety. Recently, data from a clinical care guideline aimed at improving post-endoscopy fever management showed a reduction in health care overutilization by nearly 40% (53). The guideline appropriately instructed a small subset of patients with clinically significant fever to seek medical evaluation. Abdominal pain represents another important post procedural complication and reducing discomfort with carbon dioxide insufflation has gained popularity in recent years. This technique significantly reduces post endoscopy abdominal pain within the 6 h following the procedure (54, 55) and could lead to reduced healthcare overutilization.

### Putting It Into Practice

- Endoscopy centers should develop ways to systematically record adverse events during and within the 72 h following procedures. AE data may reveal unique challenges for individual centers, and these should be used to inform quality improvement efforts.
- CO2 should be considered for pediatric endoscopy because it reduces post-procedural discomfort. This may reduce caregiver anxiety and lead to reduced emergency department overutilization.
- Institutions should develop post-procedural guidelines to triage and advise patients who develop post-procedural fever or pain as this can reduce emergency department overutilization.

## Future Directions

The future of pediatric endoscopy and endoscopy in general involves technological developments that will advance the field of gastroenterology and may gradually shift the role of an endoscopist. Artificial intelligence, robotic assistance, and disposable endoscopes are being developed to improve efficiency, diagnostic accuracy, increase procedure tolerability, and reduce the transmission of infectious disease.

AI is revolutionizing most industries because of its ability for complex data processing. Though it has been a “hot topic” for decades, AI in medicine is now taking shape largely because of the robust technological infrastructure currently in place (56). Medical education is incorporating AI into its curriculum and manufacturers are adding AI to endoscopy software (57–59). Commercially available AI software for gastroenterologists now exists through multiple manufacturers but is limited to polyp detection during endoscopy. It is reasonable to assume that with future software updates these platforms will begin to include more robust features such as identification of inflammatory lesions, and population of critical elements on a procedure report. With AI steadily on the rise, endoscopy centers interested in cutting edge technology should consider pioneering these systems within the pediatric population. AI has already been used to differentiate inflammatory lesions of the colon and diagnose celiac disease with surprising accuracy (60, 61) and to differentiate Crohn's from ulcerative colitis in pediatric patients (62). These reports indicate that AI may eventually assist physicians with real-time endoscopic diagnostic and therapeutic decision making.

AI has also shown the potential to improve the sensitivity of pill endoscopy while saving time for gastroenterologists. Recent studies involving convolutional neural networks demonstrate how computer assisted diagnosis using pill endoscopy outperforms human readers in the detection rate of pathology 88.39–99.98% vs. 74.57% and in exam completion time: 5.9 vs. 96.6 min, respectively (63, 64). AI for pill endoscopy will soon be commercially available.

Improving efficiency for clinicians is one of the most exciting improvements, but AI is not limited to high-skill tasks such as diagnostics. Documentation consumes substantial amounts of physician time and some have proposed incorporating AI into generation of procedure reports. Investigators have trained systems to recognize anatomic location, endoscopic tools, and the goodness of cleanout (65–67). Leveraging machines to generate scope report data would be a welcome opportunity for many gastroenterologists.

To address the growing concerns of exogenous infection using reprocessed endoscopes, including Carbapenem-resistant Enterobacteriaceae (68), the American Society for Gastrointestinal Endoscopy has called for enhanced endoscope reprocessing and the development of effective, environmentally friendly, disposable endoscopes (69). The first example of this is the EXALT^TM^ Model D (Boston Scientific Corporation, Marlborough, Massachusetts, USA) which achieved equal cannulation compared with standard reusable duodenoscopes in low complexity ERCPs (70). Multidrug resistant (MDR) infection is an uncommon problem in pediatrics in general. However, it is important to consider that pediatric patients undergoing ERCP within adult hospitals are at increased risk for nosocomial MDR infection spread from adult patients. Because of this risk, we suggest that adult hospitals prioritize use of disposable endoscopes in pediatric patients.

Finally, robotically assisted magnetic capsule endoscopy is an emerging technology that represents a potential paradigm shift in the way endoscopy may be performed in the future. This novel strategy employs a robotic arm wielding an electromagnet, which guides a tethered, pill-shaped endoscope through the intestine. An important patient advantage of this machine is the reduction in shearing forces on the bowel wall (71, 72). For pediatric purposes, this technology represents further miniaturization of the endoscope and could allow for the expansion of therapeutic options in very small children. This machine improves upon other magnetically guided capsules in that the tether and working channel allows for utilization of routine endoscopic tools to perform traditional diagnostic and therapeutic procedures.

### Putting It Into Practice

- Commercially available AI for endoscopy is available through multiple major manufacturers but has not been studied in pediatrics. AI for pill endoscopy will soon be available. These technologies represent an important area for future pediatric research.
- Disposable duodenoscopes are commercially available and should be considered for use in pediatric patients undergoing ERCP at adult hospitals to limit exposure to multidrug resistant bacteria.

## Conclusion

The practice of endoscopy is a cornerstone in the field of pediatric gastroenterology and has evolved over the last 50 years to include an array of advanced diagnostic and therapeutic procedures. However, despite a host of improvements, limitations related to patient safety, procedure tolerability, and diagnostic accuracy still exist. While it is not possible to know exactly how the field of pediatric endoscopy will evolve in the ensuing decades, the ongoing surge in innovation offers hope that many of today's limitations will become tomorrow's history.

## Author Contributions

CC, TL, and JK co-wrote the first draft of the manuscript and performed the literature review. GH conceived the manuscript, made significant contributions, and revised the final manuscript. All authors contributed to the article and approved the submitted version.

## Conflict of Interest

The authors declare that the research was conducted in the absence of any commercial or financial relationships that could be construed as a potential conflict of interest.
